# Molecular Evolution and Phylogeography of Co-circulating IHNV and VHSV in Italy

**DOI:** 10.3389/fmicb.2016.01306

**Published:** 2016-08-23

**Authors:** Miriam Abbadi, Alice Fusaro, Chiara Ceolin, Claudia Casarotto, Rosita Quartesan, Manuela Dalla Pozza, Giovanni Cattoli, Anna Toffan, Edward C. Holmes, Valentina Panzarin

**Affiliations:** ^1^Department of Comparative Biomedical Sciences, Istituto Zooprofilattico Sperimentale delle VeneziePadova, Italy; ^2^Charles Perkins Centre, School of Life and Environmental Sciences and Sydney Medical School, Marie Bashir Institute for Infectious Diseases and Biosecurity, University of SydneySydney, NSW, Australia

**Keywords:** VHSV, IHNV, phylogeny, evolution, molecular epidemiology

## Abstract

Infectious haematopoietic necrosis virus (IHNV) and viral haemorrhagic septicaemia virus (VHSV) are the most important viral pathogens impacting rainbow trout farming. These viruses are persistent in Italy, where they are responsible for severe disease outbreaks (epizootics) that affect the profitability of the trout industry. Despite the importance of IHNV and VHSV, little is known about their evolution at a local scale, although this is likely to be important for virus eradication and control. To address this issue we performed a detailed molecular evolutionary and epidemiological analysis of IHNV and VHSV in trout farms from northern Italy. Full-length glycoprotein gene sequences of a selection of VHSV (*n* = 108) and IHNV (*n* = 89) strains were obtained. This revealed that Italian VHSV strains belong to sublineages Ia1 and Ia2 of genotype Ia and are distributed into 7 genetic clusters. In contrast, all Italian IHNV isolates fell within genogroup E, for which only a single genetic cluster was identified. More striking was that IHNV has evolved more rapidly than VHSV (mean rates of 11 and 7.3 × 10^−4^ nucleotide substitutions per site, per year, respectively), indicating that these viruses exhibit fundamentally different evolutionary dynamics. The time to the most recent common ancestor of both IHNV and VHSV was consistent with the first reports of these pathogens in Italy. By combining sequence data with epidemiological information it was possible to identify different patterns of virus spread among trout farms, in which adjacent facilities can be infected by either genetically similar or different viruses, and farms located in different water catchments can be infected by identical strains. Overall, these findings highlight the importance of combining molecular and epidemiological information to identify the determinants of IHN and VHS spread, and to provide data that is central to future surveillance strategies and possibly control.

## Introduction

IHNV and VHSV are the causative agents of infectious haematopoietic necrosis (IHN) and viral haemorrhagic septicaemia (VHS), respectively, two OIE (World Organisation for Animal Health), listed viral diseases that severely affect trout farming (OIE, 2016). IHNV and VHSV both belong to the genus *Novirhabdovirus* within the family *Rhabdoviridae* (ICTV, 2014). These bullet-shaped, enveloped viruses possess a non-segmented, negative-sense, single-stranded RNA molecule of approximately 11 kb, that contains six genes in the order 3′-N-P-M-G-NV-L-5′, encoding the nucleocapsid protein (N), the phosphoprotein (P), the matrix protein (M), the glycoprotein (G), the non-virion protein (NV), and the polymerase protein (L) (Hoffmann et al., 2005; Kuzmin et al., 2009; Kurath, 2012).

Phylogenetic studies based on the analysis of the G and N genes showed that VHSV can be classified into four genotypes (I–IV; Einer-Jensen et al., 2004; Snow et al., 2004). Genotypes I and IV are further divided into five (Ia, Ib, Ic, Id, Ie; Einer-Jensen et al., 2004) and three (IVa, IVb, IVc; Pierce and Stepien, 2012) sublineages, respectively. VHSV genotypes have differing geographic distributions. Sublineage Ia includes freshwater isolates from continental European countries, and is further divided into two distinct sublineages: Ia1, comprising strains isolated mainly from Danish rainbow trout, and Ia2, representing viruses that predominantly originate in continental Europe. Sublineage Ib circulates in marine fish of the Baltic and North Sea and the English Channel, while clade Ic comprises Danish rainbow trout strains from the 1980s. Sublineages Id and Ie include viral isolates from the Scandinavian peninsula and from the Black Sea, respectively, while viral strains within genotypes II and III appear to be restricted to the Baltic Sea and the North Atlantic Ocean. Finally, genotype IV has been reported in North America and Asia. Similarly, analysis of G gene sequences of IHNV isolates revealed the presence of five major “genogroups” termed U (upper), M (middle), L (lower), E (European), and J (Japanese) (Kurath, 2012). The designation U, M, and L reflects the geographical distribution of IHNV in North America (Kurath et al., 2003; Kuzmin et al., 2009; Enzmann et al., 2010), while genogroup E is related to the M genogroup and comprises European isolates from France, Italy and Germany (Enzmann et al., 2005). Finally, genogroup J includes rainbow trout isolates from Japan and South Korea (Nishizawa et al., 2006; He et al., 2013).

Italy is the top producer of rainbow trout (*Oncorhynchus mykiss*) in Europe, yielding about 34.400 tons live weight/year (Food and Agriculture Organization [FAO], 2014). Trout husbandry started in Italy in the 1950s, and today accounts for approximately 66% of the national finfish production, with nearly 500 farms overall that are largely located in the north of the country. Although, this business yields approximately 114 million USD annually (Food and Agriculture Organization [FAO], 2014), the profitability of the trout industry is threatened by both VHS and IHN which were reported in Italy for the first time in 1960 and 1987, respectively (Ghittino, 1965; Bovo et al., 1987). Since then fish farmers and local authorities have made a major effort to control and prevent the spread of the infection. However, despite compulsory surveillance programs and a European directive, VHS and IHN still represent a major threat to Italian trout farming. To develop efficient disease control strategies, it is essential to determine the evolution of VHSV and IHNV, and to integrate molecular and epidemiological data to trace the origin and the spread of these viruses. Within this framework, molecular epidemiology represents a powerful tool that can provide key information on the evolution, ecology and transmission pathway of fish viral pathogens, which may then be effectively translated into disease control policies.

To reveal the evolution and the epidemiology of IHN and VHS in Italy we analyzed, using phylogenetic, statistical and visual methods, a representative collection of viral strains isolated in the northeastern part of the country during natural outbreaks of both diseases over the past 30 years. This enabled us to greatly broaden our knowledge of the evolution and epidemiology of these two economically important viruses, including the development of hypotheses for the different possible routes of viral spread among trout farms. In turn, this information may assist in the development of intervention strategies to reduce any future incidence of these major agricultural diseases.

## Materials and methods

### Ethics statement

No animal experiments were performed during this study. Viral strains were isolated from fish specimens derived from the diagnostic and monitoring activities carried out at the National Reference Centre for Fish, Crustacean and Mollusc Pathology hosted by the Istituto Zooprofilattico Sperimentale delle Venezie (IZSVe). Surveillance, both active and passive, was performed by competent local veterinary authorities according to the relevant legislation (Directive 91/67/EC; Directive 2006/88/EC) through the periodical inspections of farms as well as samples collection in case of clinical suspicion.

### Viral strains and sampling area

**Figure 1:**
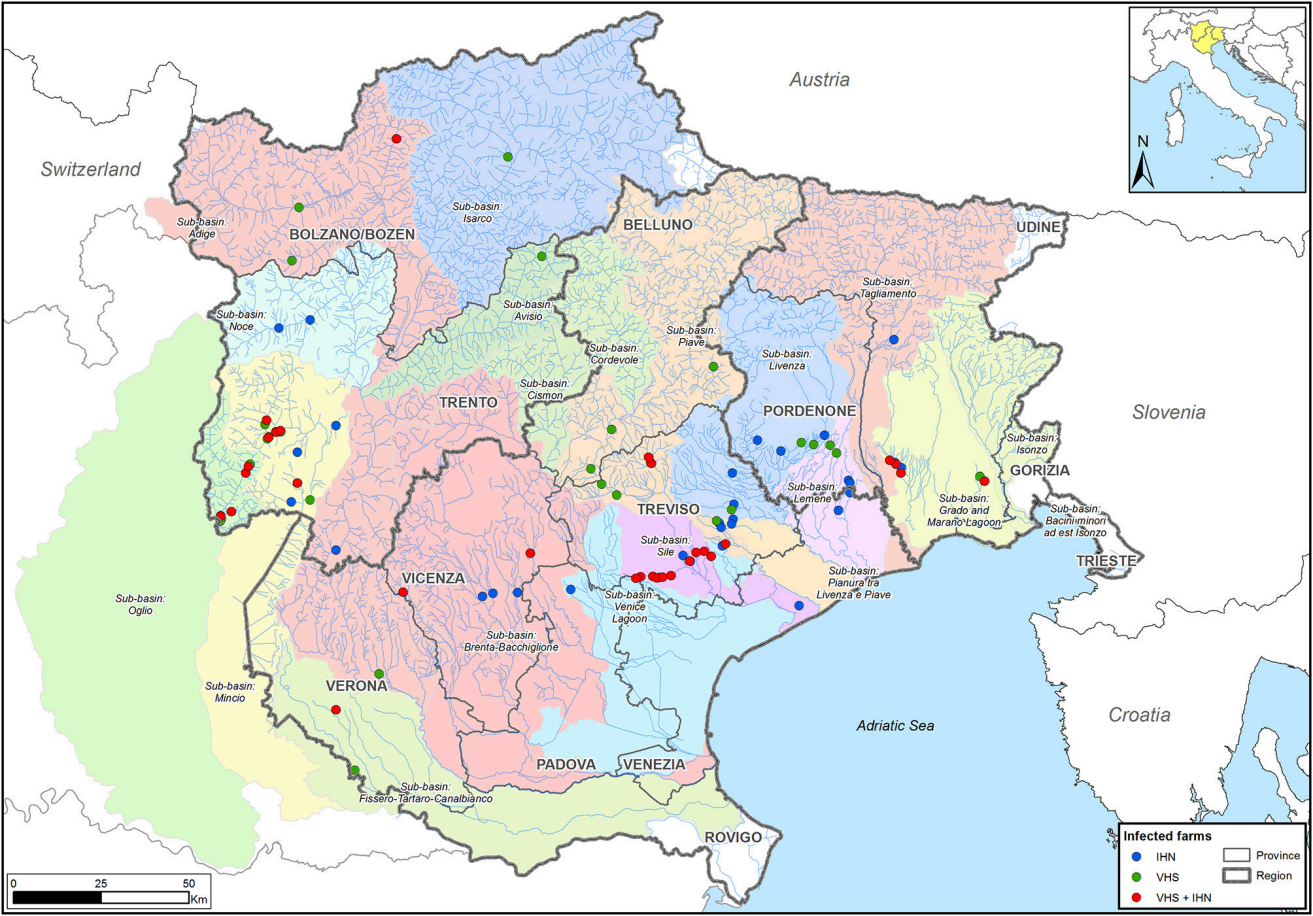
**Sampling area**. The infected farms studied here are located in the northeastern part of Italy, in Trentino Alto Adige, Veneto, and Friuli Venezia Giulia regions. Farms are represented by dots colored according to the disease etiology (blue: IHN; green: VHS; red: IHN + VHS).

The strains of IHNV (*n* = 89) and VHSV (*n* = 108) were selected to cover the broadest spatio-temporal range. A total of 16 of the 197 samples were IHNV/VHSV co-infections. Viral isolates originate from different rainbow trout farms in the northeastern part of Italy, within 14 different water sub-basins. The sampling area covers the regions of Friuli Venezia Giulia, Trentino Alto Adige and Veneto, which account for 65% of the Italian fresh-water salmonid production and comprise approximately 200 facilities. All in all, 61 and 62 farms were investigated for IHN and VHS, respectively (Figure 1), for a total number of 93 facilities. Overall, selected isolates cover a time period between 1982 and 2013. IHNV and VHSV strains were previously isolated using *epithelioma papulosum cyprini* (EPC) and/or bluegill fry (BF-2) cell lines and originated predominantly from rainbow trout (*O. mykiss*) specimens, with the exception of 8 samples from brook trout (*Salvelinus fontinalis*), brown trout (*Salmo trutta*), Northern pike (*Esox lucius*) and redfin perch (*Perca fluviatilis*).

### RNA extraction, RT-PCR, and sequencing

Total RNA was extracted from 100 μl of cell culture supernatant using NucleoSpin RNA II (Macherey–Nagel GmbH & Co., Düren, Germany) according to the manufacturer's instructions. Reverse transcription followed by PCR amplification of the complete G gene was performed with the Qiagen OneStep RT-PCR kit (Qiagen GmbH, Hilden, Germany). The primers used are reported in Table 1. The reverse transcription and the amplification reaction were carried out in 50 μl of reaction volume for VHSV and in 25 μl for IHNV. The reaction mix contained 0.6 μM of each primer, 0.4 mM dNTPs, 1X PCR buffer, and 0.5 μM of OneStep RT-PCR Enzyme Mix. The applied thermal conditions were as follows: 50°C for 30 min, 95°C for 15 min and 35 cycles of 1 min denaturation at 94°C, 30 sec annealing at 56°C (IHNV) or 58°C (VHSV) and 1 min (IHNV) or 2 min (VHSV) elongation at 72°C; the reaction was terminated with 10 min elongation at 72°C.

**Table 1:** **Primers used for the amplification and sequencing of the IHNV and VHSV full-length G gene**.

**Primer**	**Sequence 5′→3′**	**Position**	**Use**	**Amplicon size (bp)**	**References**
**VHSV**					
GB+	GTCGAAGAAGAGATAGGC	2796-2813^a^	RT-PCR	1171 bp	Einer-Jensen et al., 2004
GB-	GTTGGGTCGCCATGTTTCT	4548-4566^a^			Einer-Jensen et al., 2004
VHS Seq10 m-F	CCCTGGGCCTGGCAA	3975-3989^a^	Sequencing	–	This study
VHS Seq10 m-R	TTGCCAGGCCCAGGG	3975-3989^a^	Sequencing	–	This study
GSeq2+	GCCCATTGCCCCACG	3592-3606^a^	Sequencing	–	Einer-Jensen et al., 2004
VHS Seq2-R	CGTGGGGCAATGGGC	3592-3606^a^	Sequencing	–	This study
GSeq4+	CCTTGTGGAAGTCCCTC	4227-4243^a^	Sequencing	–	Einer-Jensen et al., 2004
VHS Seq4-R	GAGGGACTTCCACAAGG	4227-4243^a^	Sequencing	–	This study
GSeq6-	GCACAGAGTGACTTATCG	3258-3275^a^	Sequencing	–	Einer-Jensen et al., 2004
VHS Seq6-R	CGATAAGTCACTCTGTGC	3258-3275^a^	Sequencing	–	This study
**IHNV**					
IHNVfl-FOR	CTCACTCCGTCCAAGACAG	2928–2946^b^	RT-PCR/Sequencing	782 bp	This study
IHNV-Rev1	CCTTCACGRCYCGATTGGAG	3690–3709^b^	RT-PCR/Sequencing		
G1 FOR	AGAGATCCCTACACCAGAGAC	3523–3543^b^	RT-PCR/Sequencing	499 bp	
IHNV-REV2	GATGTGGAGAKCGGAACTTG	4002–4021^b^	RT-PCR/Sequencing		
IHNV IGSeq 5-F	GCACGCCGAGATAATATC	3954–3971^b^	RT-PCR/Sequencing	721 bp	
IHNVfl-REV	GCCACCTTGTTCTTGTATC	4656–4674^b^	RT-PCR/Sequencing		

*Sequence, position of binding to the reference sequence, use and amplicon size are reported*.

a *Nucleotide positions where primers bind are in accordance with the sequence of VHSV strain 07–71 under the GenBank accession number AJ233396*.

b *Nucleotide positions where primers bind are in accordance with the IHNV sequence under the GenBank accession number X89213*.

PCR products were analyzed for purity and size by electrophoresis in 1.5% agarose gel (Sigma-Aldrich, St. Louis, MO) after staining with 0.1 μl/ml of GelRedTM Nucleic Acid Gel Stain (Biotium, Hayward, CA). Amplicons were subsequently purified with ExoSAP-IT (USB Corporation, Cleveland, OH) and sequenced in both directions using the Big Dye Terminator v3.1 cycle sequencing kit (Applied Biosystems, Foster City, CA). Sequencing reactions were cleaned-up using the Performa DTR Ultra 96-well kit (Edge BioSystems, Gaithersburg, MD) and analyzed on a 16-capillary ABI PRISM 3130xl Genetic Analyzer (Applied Biosystems, Foster City, CA, USA).

### Nucleotide sequence accession numbers

All IHNV and VHSV G gene sequences generated here have been deposited on GenBank and assigned accession numbers KU878165-KU878361.

### Phylogenetic analysis

Sequence data were assembled and edited using the SeqScape software v2.5 (Applied Biosystems). The consensus G gene sequences obtained were aligned and compared to reference sequences retrieved from GenBank using the MEGA 5 software (Tamura et al., 2011). To infer the phylogenetic relationships among the isolates we used the maximum likelihood (ML) method available in the PhyML program version 3.1 (Guindon et al., 2010), incorporating a general time-reversible (GTR) model of nucleotide substitution with a gamma-distribution of among-site rate variation (with four rate categories, Γ_4_) and a SPR branch-swapping search procedure (Darriba et al., 2012). To assess the robustness of individual nodes, 1000 bootstrap replicates were performed using the same substitution model as described above. Phylogenetic trees were visualized with the FigTree v1.4 software (http://tree.bio.ed.ac.uk/software/figtree/). The amino acid mutations fixed along branches of the phylogenies were identified using the parsimony algorithm available in the Mesquite program v3.04 (Maddison and Maddison, 2014).

### Analysis of selection pressures

For both IHNV and VHSV data sets, the gene and site-specific selection pressures acting on the sequences were estimated as the ratio of non-synonymous (*d*_N_) to synonymous (*d*_S_) nucleotide substitutions per site. Accordingly, *d*_N_/*d*_S_ ratios were assessed using the single-likelihood ancestor counting (SLAC), the fixed-effects likelihood (FEL), the internal fixed-effects likelihood (IFEL) and the fast unconstrained bayesian approximation (FUBAR) methods (Pond and Frost, 2005; Murrell et al., 2013) available at the Datamonkey online version of the Hy-Phy package (Delport et al., 2010). These analyses utilized the GTR model of nucleotide substitution and employed input neighbor-joining phylogenetic trees.

### Nucleotide substitution rates and the time-scale of virus evolutionary history

To initially assess the extent of a temporal structure in IHNV and VHSV data sets, a necessary prerequisite for the reliable estimation of substitution rates, we performed a regression of root-to-tip genetic distances against sampling date using the TempEst program (Rambaut et al., 2016). The analyses were based on the ML phylogenetic trees as input.

Rates of nucleotide substitution per site, per year and the time to the most recent common ancestor (tMRCA) were estimated for both data sets using the Bayesian Markov Chain Monte Carlo (MCMC) method available in BEAST, version 1.8.1 (Drummond and Rambaut, 2007). A HKY85 + Γ_4_ model of nucleotide substitution with two data partitions of codon positions (1st + 2nd positions, 3rd position) was used, with base frequencies unlinked across all codon positions through the SRD06 substitution model. The performance of the marginal likelihood estimators, specifically, path sampling (PS) and stepping-stone (SS) sampling (Baele et al., 2012) were assessed to compare and select the best fitting molecular clock model (uncorrelated lognormal relaxed clock vs. strict clock). The Bayesian skyride coalescent was applied as a tree prior, and a gamma distribution (initial value 0.001, shape 0.001, scale 1000) was set as a nucleotide substitution rate prior. Statistical uncertainty is reflected in values of the 95% highest probability density (HPD) for each parameter estimate. Chain lengths were run for 50 million iterations to achieve convergence as assessed using Tracer v1.5 (Drummond and Rambaut, 2007). Maximum Clade Credibility (MCC) phylogenetic trees were summarized from the posterior distribution of trees using TreeAnnotator v1.6.1 (Drummond and Rambaut, 2007) after the removal of an appropriate burn-in (10% of the samples). The MCC trees were visualized using the program FigTree v1.4.

### Phylogeny-trait association analysis and epidemiological investigations

To test the null hypothesis that the phylogenetic relationships of the Italian IHNV and VHSV is uncorrelated with their geographic origin (i.e., no stronger than by chance alone), we grouped the sequences according to their water basin of origin and used the Bayesian tip-association significance testing (BaTS beta build 2) program (Parker et al., 2008) to estimate values of the association index (AI) and parsimony score (PS) statistics of phylogeny-trait association, with the trait (the basin of origin) as defined above. For this purpose the IHNV viruses were grouped into 10 different water catchments: Adige (comprising Adige and Noce sub-basins), Brenta, Grado-Marano, Lemene, Livenza, Piave, Po (including Mincio and Oglio sub-basins), Sile, Tagliamento, and Venice Lagoon. Similarly, VHSV strains were distributed within 9 diverse basins of origin: Adige (comprising Adige, Avisio and Isarco sub-basins), Brenta, Fissero-Tartaro-Canal Bianco, Grado-Marano, Lemene, Livenza, Piave, Po (including Mincio and Oglio sub-basins) and Sile. BaTS accounts for phylogenetic uncertainty in the data by using the posterior distribution of trees obtained from the BEAST analyses previously described. We also assessed the level of clustering in individual water basins using the monophyletic clade (MC) size statistic (Parker et al., 2008). For each analysis, 2000 random permutations of tip locations were undertaken to create a null distribution. To be as conservative as possible, only results with *P* < 0.01 were considered to be statistically significant.

To reconstruct the transmission pathways of IHNV and VHSV among diverse trout farms located in the same river or in different water catchments in more detail, sequence data and epidemiological data relating to the disease outbreaks were integrated in a more rigorous manner considering arbitrary time slots from five to seven years (i.e., 1991–1995, 1996–2001, 2002–2006, and 2007–2013). The epidemiological data gathered included the date of sampling, the farm of origin, the river catchment, the water source (river, spring or well) and the water discharge. The geographical coordinates of the farms were also uploaded, together with all the above mentioned information, into a geodatabase. To investigate the patterns of spread of IHNV and VHSV among farms, a visual analysis was performed using ArcGIS V10.0 (http://www.esri.com/software/arcgis/arcgis-for-desktop). Infected farms were labeled on a map in different colors in accordance with the assignment of the IHNV and VHSV strains to the genetic groups identified in the phylogenetic analysis. More details on the relevant epidemiological information are given in Table 2.

**Table 2:** **Epidemiological information of the viral strains used to infer transmission pathways in IHNV and VHSV**.

**Sample ID**	**Accession number**	**Sampling date**	**Co-infection**	**Genetic group**	**Sub-basin**	**Water source**	**Drain: River**
**IHNV STRAINS**							
IHNV/O.mykiss/I/TV/299/Jun03 (8)	KU878325	17/06/2003	NO	A	Sile	R, S, W	YES
IHNV/O.mykiss/I/TV/151/Apr04 (10)	KU878330	22/04/2004	NO	A	Sile	R, S, W	YES
IHNV/O.mykiss/I/TV/310/May05 (9)	KU878338	30/05/2005	NO	A	Sile	S, W	YES
IHNV/O.mykiss/I/TV/459/Sep05 (11)	KU878339	02/09/2005	NO	A	Sile	R	YES
IHNV/O.mykiss/I/TV/225/Jun07 (22)	KU878348	08/06/2007	YES (VHSV)	A	Sile	S, W	YES
IHNV/O.mykiss/I/TV/234/May08 (21)	KU878350	15/05/2008	NO	A	Sile	R, S, W	YES
IHNV/O.mykiss/I/TV/459/Oct08 (24)	KU878352	28/10/2008	NO	non A	Sile	R, S, W	YES
IHNV/O.mykiss/I/TV/21/Jan12 (23)	KU878357	19/01/2012	YES (VHSV)	non A	Sile	R, S, W	YES
**VHSV STRAINS**							
VHSV/O.mykiss/I/UD/670/Nov95 (27)	KU878178	24/11/1995	NO	C	Grado e Marano	R, S, W	YES
VHSV/O.mykiss/I/TV/673/Nov95 (25)	KU878179	30/11/1995	NO	C	Sile	S, W	YES
VHSV/O.mykiss/I/PN/708/Dec95 (26)	KU878180	14/12/1995	NO	C	Lemene	R	YES
VHSV/O.mykiss/I/TN/510/Nov03 (5)	KU878208	25/11/2003	NO	F1	Mincio	R	YES
VHSV/O.mykiss/I/TN/511/Nov03 (7)	KU878209	25/11/2003	NO	F1	Mincio	R, S, W	YES
VHSV/O.mykiss/I/TN/537/Dec03 (6)	KU878211	02/12/2003	NO	F1	Mincio	R	YES
VHSV/O.mykiss/I/TN/316/May05 (3)	KU878230	31/05/2005	NO	F1	Mincio	R	YES
VHSV/O.mykiss/I/TN/616/Nov05 (4)	KU878235	09/11/2005	NO	F1	Mincio	R	YES
VHSV/O.mykiss/I/TN/750/Dec05 (2)	KU878236	20/12/2005	NO	E	Oglio	R	YES
VHSV/O.mykiss/I/TN/9/Jan06 (1)	KU878237	12/01/2006	NO	E	Oglio	R, S, W	YES
VHSV/O.mykiss/I/TN/182/Apr08 (20)	KU878248	24/04/2008	NO	F1	Mincio	R, S, W	YES
VHSV/S.trutta/I/TN/470/Nov09 (19)	KU878251	24/11/2009	NO	A	Mincio	R	YES
VHSV/O.mykiss/I/TN/133/Apr10 (12)	KU878254	06/04/2010	NO	F1	Mincio	R	YES
VHSV/O.mykiss/I/TN/237/May10 (17)	KU878256	18/05/2010	NO	F1	Mincio	S, W	YES
VHSV/O.mykiss/I/TN/8/Jan11 (16)	KU878260	18/01/2011	NO	F1	Mincio	R	YES
VHSV/O.mykiss/I/TN/28/Feb11 (13)	KU878262	08/02/2011	NO	F1	Mincio	R	YES
VHSV/O.mykiss/I/TN/89/Mar11 (15)	KU878264	29/03/2011	NO	F1	Mincio	R	YES
VHSV/O.mykiss/I/TN/124/Apr11 (18)	KU878268	12/04/2011	NO	F1	Mincio	R	YES
VHSV/O.mykiss/I/TN/106/Feb12 (14)	KU878270	21/02/2012	NO	F1	Mincio	R	YES

*The numbers in parentheses following the Sample ID refer to the farm designation as shown in Figure 4. The following data are reported for each sample: GenBank accession number, sampling date, the occurrence of co-infection, the membership to the genetic groups identified, the sub-basin, the water source (R, River; S, Spring; W, Well) and the water discharge*.

## Results

### Phylogenetic analysis

**Figure 2:**
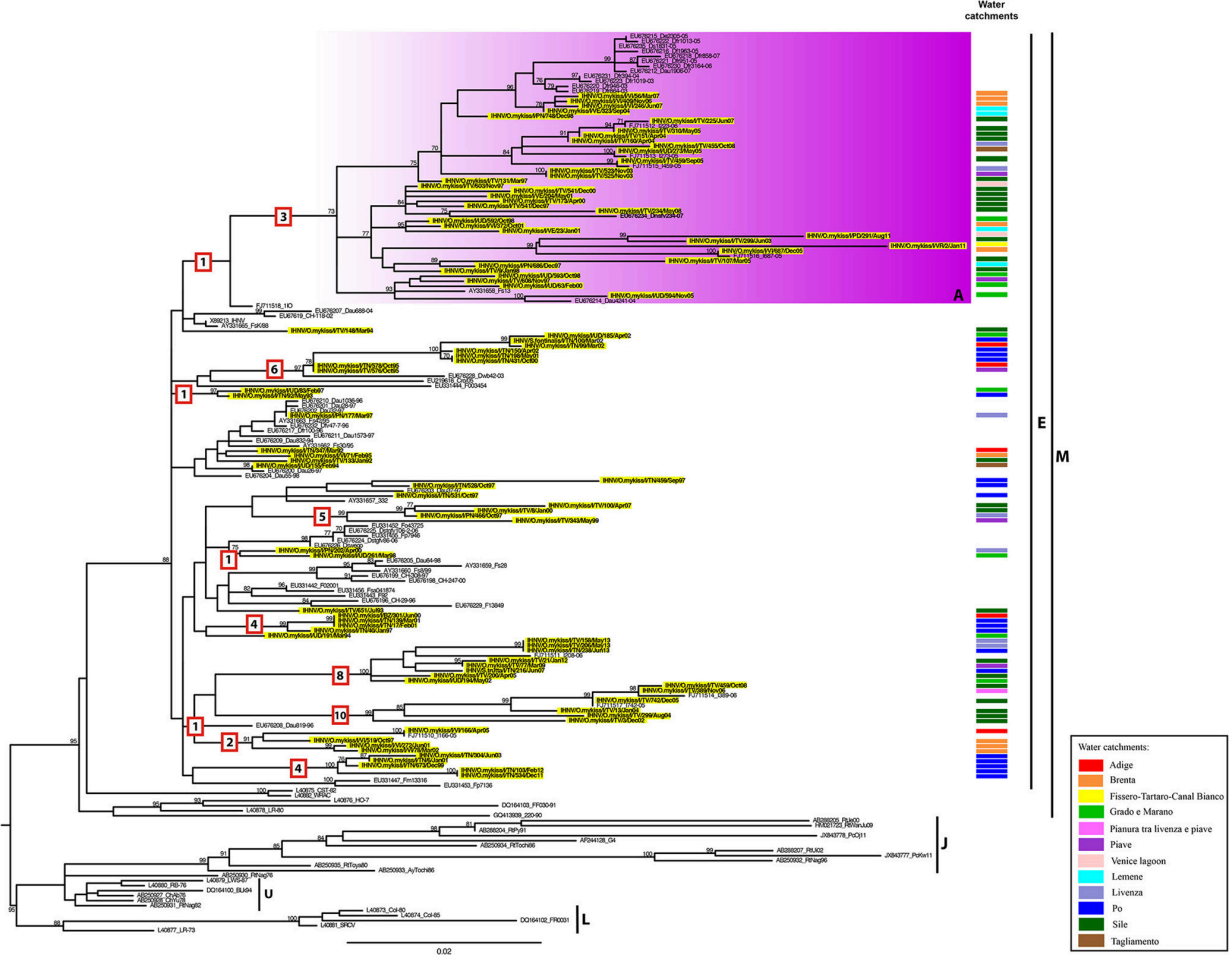
**ML phylogenetic tree of IHNV based on the complete G gene**. The Italian strains are highlighted in yellow and labeled according to their water basin of origin as indicated in the “Water catchments” column. IHNV genotype subdivision is designated by vertical bars. The colored box represents the identified genetic group (A) within genogroup E. For the Italian strains under investigation, the water basin of origin has been indicated. The numbers at nodes represent bootstrap values (only values >70% are reported), while branch lengths are scaled according to the number of nucleotide substitutions per site. The scale bar is reported. The numbers within the red boxes represent the amino acid substitutions occurring along the branches. The tree is mid-point rooted for clarity only.

A maximum likelihood phylogenetic analysis reveals that all the Italian IHNV strains fall within the European genogroup E (Figure 2). In this phylogeny we identified one main monophyletic group including 35 Italian viruses and defined by a bootstrap value of 73% (group A). This group comprises IHNV strains originating from the Veneto and Friuli Venezia Giulia regions, isolated between 1997 and 2011. In addition, several small genetic groups composed of 2–9 Italian sequences, to which no name has been assigned, were also observed within genogroup E (Figure 2). Whether these clusters represent independent introductions of the virus into Italy or the parallel evolution of separate viral lineages, requires further investigation. The Italian strains belonging to genogroup E are related to IHNV strains from Croatia, France, Germany and Switzerland. Interestingly, the amino acid similarity estimated among Italian IHNV strains is slightly lower than that calculated for Italian VHSV (100–93.9 vs. 100–95.3%; see below). This is consistent with the greater number of fixed amino acids mutations along branches observed for IHNV (Figure 2).

**Figure 3:**
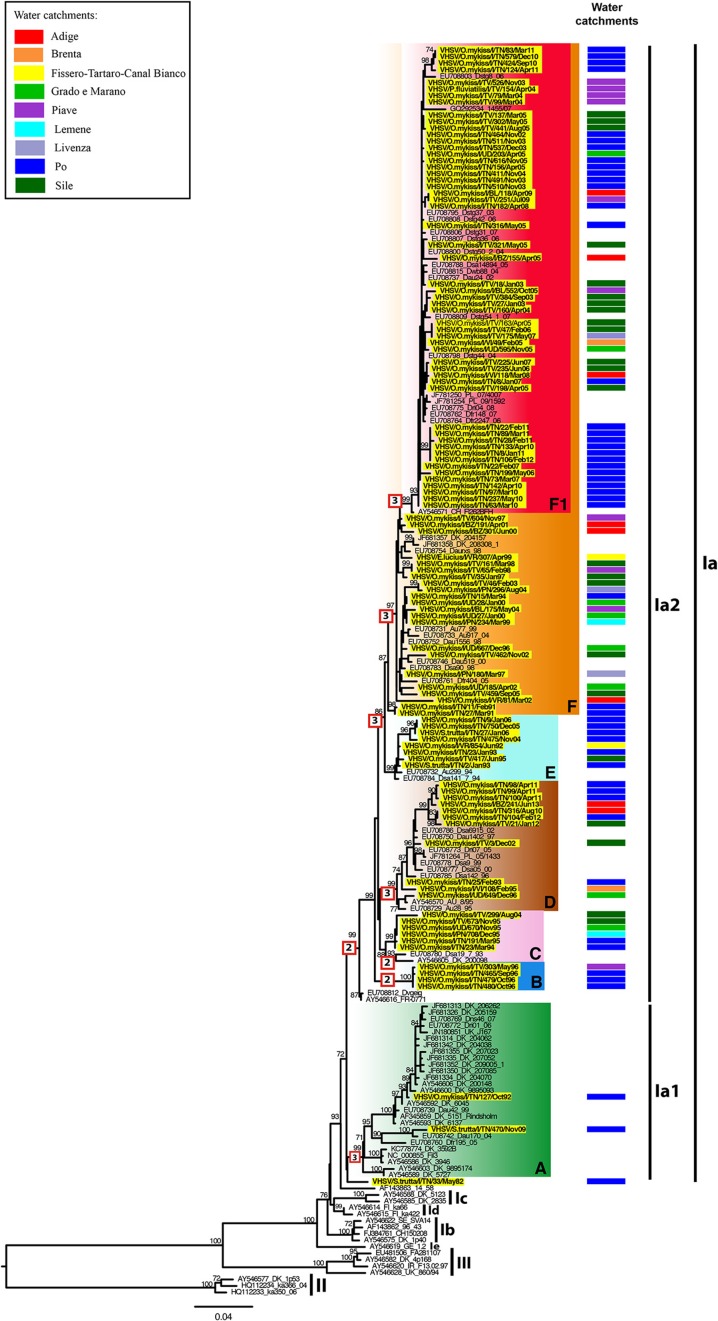
**ML phylogenetic tree of VHSV based on the complete G gene**. The Italian strains are highlighted in yellow and labeled according to their water basin of origin as indicated in the “Water catchments” column. VHSV genotype subdivision is designated by vertical bars. The colored boxes represent the 7 different genetic clusters (A,B,C,D,E,F,F1) identified within genotype Ia, sublineages Ia1 and Ia2. For the Italian strains under investigation, the water basin of origin has been indicated. The numbers at nodes represent bootstrap values (only values >70% are reported), while branch lengths are scaled according to the number of nucleotide substitutions per site. The scale bar is reported. The numbers within the red boxes represent the amino acid substitutions occurring along the branches. The tree is mid-point rooted for clarity only.

The VHSV phylogeny indicates that the Italian VHSV viruses belong to sublineage Ia, and are further divided into clades Ia1 (*n* = 105) and Ia2 (*n* = 2) (Figure 3), indicative of multiple introductions of VHSV in the country. The tree topology is characterized by the existence of seven genetic groups defined by high bootstrap values (>70%) arbitrarily named A, B, C, D, E, F and F1. Notably, each genetic cluster includes viral strains originating from different water catchments. Group A comprises viruses from Denmark, Germany and United Kingdom, as well as two strains isolated in 1992 and 2009 in Italy. Group B only includes Italian viruses isolated in 1996, while group C contains strains from Italy detected between 1994 and 2004, and viruses from Denmark and Germany. Group D contains isolates collected in the period 1993–2013 in Italy in addition to Austrian, German and Polish strains. Cluster E comprises viruses isolated in Italy in the time periods 1992–1995 and 2004–2006, and in 1994 in Austria and Germany. Finally, group F, and its derived group F1, contain strains collected from year 1991 in Italy, as well as viruses from Austria, Denmark, Germany, Poland, Slovenia, and Switzerland. Notably, group F1 comprises only Italian recent strains (2002–2012). Sample VHSV/S.trutta/I/TN33/May82 appears to be the basal member of the Ia sublineage.

### Analysis of selection pressures

Most codons in the G genes of Italian IHNV and VHSV were identified as subject to purifying selection, with mean *d*_N_*/d*_S_ ratios of 0.38 and 0.21, respectively. However, a number of codons in the G gene may be subject to positive selection (*P* ≤ 0.05; posterior probability ≥ 0.9). Specifically, nine sites were identified as positively selected in IHNV, positions 24, 98, 232, 247, 252, 276, 277, 286, and 475. Of these, four (24, 247, 252, and 277) were confirmed in all the methods used here (SLAC, FEL, IFEL, and FUBAR), while the other five sites were identified by at least two of the methods applied (Table 3). For VHSV, a total number of four sites putatively subject to positive selection, at positions 212, 258, 259, and 290 were identified. Sites 258 and 259 were confirmed by all four models, while sites 212 and 290 were supported by two different models (Table 3).

**Table 3:** **Amino acid sites under putative positive selection in the Italian IHNV and VHSV strains**.

**Virus**	**Site**	**SLAC**	**FEL**	**IFEL**	**FUBAR**
		***P* ≤ 0.05**			**Posterior Probability ≥ 0.9**
VHSV	212		0.040		0.99
	258	0.005	0.0004	0.0006	0.99
	259	0.033	0.044	0.042	0.99
	290			0.035	0.90
IHNV	24	0.039	0.006	0.001	0.99
	98			0.027	0.92
	232		0.042		0.97
	247	0.019	0.004	0.0006	0.99
	252	0.009	0.003	0.001	0.99
	276		0.049	0.027	0.96
	277	0.011	0.010	0.029	0.99
	286		0.030	0.020	0.98
	475		0.041	0.039	

*Different analytical models (SLAC, FEL, IFEL, and FUBAR) were applied*.

### Rates and dates of IHNV and VHSV evolution

Our root-to-tip regression of the viral G gene revealed relatively strong temporal structure in both IHNV and VHSV, with correlation coefficients of 0.763 and 0.859, respectively. Such a relationship between genetic distance and time enables more detailed analyses of evolutionary dynamics. Indeed, using this simple regression method we estimated evolutionary rates of 9.9 × 10^−4^ and 6.6 × 10^−4^ subs/site/year for IHNV and VHSV, respectively.

We next inferred the evolutionary rates of the G gene using a Bayesian coalescent approach (Drummond and Rambaut, 2007). The mean evolutionary rate of the Italian IHNV strains was estimated to be 11 × 10^−4^ subs/site/year (95% HPD, 9.1 × 10^−4^ – 13 × 10^−4^ subs/site/year). In contrast, the rate of nucleotide substitution estimated for Italian VHSV was 7.3 × 10^−4^ subs/site/year (95% HPD, 5.8 × 10^−4^ – 8.9 × 10^−4^ subs/site/year). The lack of overlap between the HPD values suggests that IHNV has evolved significantly more rapidly than VHSV. These rates were also used to estimate times to common ancestry. Accordingly, the tMRCA calculated for the Italian IHNV and VHSV data sets date back to 1985 (95% HPD 1982–1988) and 1974 (95% HPD 1968–1979), respectively.

### Phylogeography and visual spatial analysis

The BaTS analysis of phylogeny-trait associations revealed no statistically significant geographic structuring of viruses by water basin (*P* ranges between 0.01 and 1 for both data sets; Table S1), with the exception of the Po basin which showed a *P* ≤ 0.001 for both IHNV and VHSV, and the Brenta basin displaying a *P* = 0.004 for IHNV. This lack of a geographical clustering for most of the viruses can be observed also in the phylogenetic trees annotated according to the basin of origin (Figures 2, 3).

The visual reconstruction of the transmission pathways of IHNV and VHSV among diverse trout farms allowed us to identify three different diffusive patterns for viral spread.

**Figure 4:**
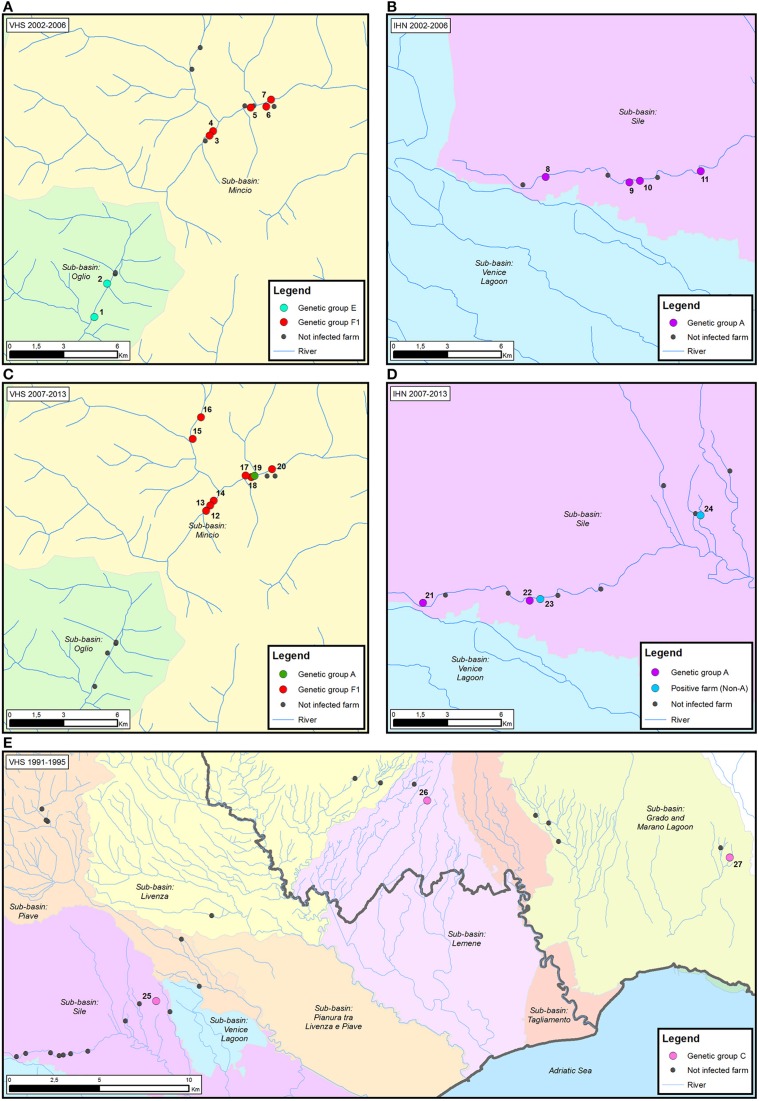
**Examples of the proposed patterns of IHNV and VHSV spread in Italy**. (i) neighboring farms located in the same water system experiencing disease outbreaks caused by genetically related strains **(A,B)**; (ii) genetically diverse viruses in neighboring farms **(C,D)**; and (iii) infection by identical viruses in farms with no connection via water **(E)**. The time period considered is reported for each map. Infected trout farms are represented by dots colored according to the assignment of the IHNV and VHSV strains to the genetic groups identified in the phylogenetic analysis. Non-infected trout farms are designated in black. The number associated to each infected farm corresponds to the isolates reported in Table 2.

First, neighboring farms located in the same water system experienced disease outbreaks caused by genetically related strains. For example, in the case of VHSV in the period 2002–2006 two trout farms (farms 1–2) located in the sub-basin Oglio were infected with genetically identical viruses belonging to the E group (Figure 4A). Similarly, viral strains belonging to group F1, and sharing 99.9–100% nucleotide identity, infected five different fish farms all situated in the sub-basin Mincio (farms 3–7). The same situation was observed for IHNV, where four different trout farms (farms 8–11), all located in the proximity of the river Sile, experienced the infection of viral strains belonging to the genetic group A (99.8–97% nucleotide identity) in the period 2002–2006 (Figure 4B). Notably, all the farms under investigation took the water and released it back into the same river, with the exception of farm 9 which procures water from well or spring.

A second pattern of virus spread marks the occurrence of genetically diverse viruses in neighboring farms. In the time period from 2007 to 2013, eight out of nine farms located in the sub-basin Mincio (farms 12–18, 20) experienced the infection of VHSV strains belonging to the F1 genetic group, while in farm 19 the disease outbreak was caused by a viral strain belonging to group A (Figure 4C). Similarly, in the same time period, genetically different IHNV strains (97.1–95.4% identity) were isolated in four neighboring farms (farms 21–24) situated in the sub-basin Sile (Figure 4D). Two out of four viruses detected in farms 21 and 22 belong to genetic group A, while the remaining two strains were identified as “non-A.” The water source for all the above mentioned farms consists of river, well and spring water, with the exception of farms 17 and 22 that use only spring water.

Finally, in the third spread pattern, we observed infection by identical viruses in farms with no connection via water. For example, in the case of VHSV during the period 1991–1995, identical viruses belonging to group C were detected in farms 25, 26, and 27, situated in the sub-basins Sile, Lemene, and Grado-Marano Lagoon, respectively (Figure 4E). A similar pattern was observed in IHNV, where identical viruses belonging to group A infected farms located in different water sub-basins (distribution map not shown).

## Discussion

Viral infectious haematopoietic necrosis (IHN) and viral haemorrhagic septicaemia (VHS) represent a severe threat to the sustainability and the profitability of Italian trout farming. The phylogenetic study of their causative agents, as well as the elucidation of the ecological, evolutionary and epidemiological processes characterizing new disease outbreaks, provides valuable information on the mechanisms of viral emergence and spread. Importantly, such information could also assist the central and local veterinary authorities in defining containment areas and in developing effective virus eradication programs (see Decision 2015/1554/EC).

We performed the first large-scale phylogenetic and evolutionary analysis of Italian IHNV and VHSV based on the complete glycoprotein sequence. Although these pathogens share a number of ecological features including their spatio-temporal distribution and the species tropism, which explains the high number of co-infected hosts, they exhibited markedly different evolutionary patterns. Specifically, Italian VHSV strains can be divided into 7 well supported genetic groups and are distributed within sublineages Ia1 and Ia2 (Kahns et al., 2012), suggesting the occurrence of different introductions of VHS in Italy. In contrast, all the Italian IHNV fell into a single genetic group—genogroup E. Within this group, we identified one main genetic cluster (group A) and several small subgroups that are characterized by a higher number of amino acid changes compared to the VHSV groups. It remains to be determined whether this is the effect of new viral introductions from external sources or if it reflects the emergence of diverse co-circulating lineages in the geographic area under investigation.

VHSV and IHNV also differ markedly in evolutionary dynamics. Specifically, IHNV has evolved more rapidly than VHSV (mean rates of 11 × 10^−4^ subs/site/year and 7.3 × 10^−4^ subs/site/year, respectively). Broadly similar rates of nucleotide substitution have been reported also by other authors for IHNV (8.71 × 10^−4^ subs/site/year, He et al., 2013; 12 × 10^−4^, Troyer and Kurath, 2003) and VHSV (17 × 10^−4^ subs/site/year, Einer-Jensen et al., 2004; 5.91 × 10^−4^ subs/site/year, He et al., 2014). While rainbow trout VHSV became established in continental Europe through several host switch events from its marine ancestor (Einer-Jensen et al., 2004; Skall et al., 2005), it has been speculated that IHNV was introduced in European trout farms through contaminated trout eggs from North America (Kurath, 2012). However, it is unclear whether these differences could have determined diverse evolutionary rates for IHNV and VHSV.

Our analyses also indicate that the common ancestor of IHNV existed between 1982 and 1988, which coincides with the first documentation of the disease in Italy in 1987 (Bovo et al., 1987). In the case of VHSV, the tMRCA was estimated to range between 1968 and 1979, although VHS was notified for the first time in Italy in 1960 (Ghittino, 1965). This discrepancy might be attributable to a sampling bias due to the unavailability in our repository of VHSV isolates older than 1982.

In agreement with other authors (Troyer and Kurath, 2003; Pierce and Stepien, 2012; He et al., 2013, 2014), the *d*_N_*/d*_S_ ratios obtained indicate that the purifying selection is the main force shaping the evolution of IHNV and VHSV. Despite this, nine and four codons were identified as subject to positive selection within the complete glycoprotein gene of the Italian IHNV and VHSV, respectively. Interestingly, amino acids at positions 24, 247, and 252 for IHNV and 258 for VHSV were identified as being positively selected in other studies (Troyer and Kurath, 2003; La Patra et al., 2008; Padhi and Verghese, 2008; He et al., 2013, 2014). In this context it is noteworthy that IHNV sites 232 and 276, and VHSV sites 258 and 259 fall within antigenic regions previously determined by neutralizing monoclonal antibodies (Huang et al., 1994, 1996; Kim et al., 1994; Béarzotti et al., 1995; Thiéry et al., 2002), suggesting a structural similarity between IHNV and VHSN glycoproteins.

Taken together, these data indicate that IHNV and VHSV experienced different evolutionary patterns. Specifically, IHNV is characterized by a higher substitution rate than VHSV and experiences stronger positive selective pressure; hence it is possible that the higher evolutionary rate is in part a function of adaptive evolution (Dixon et al., 2016). Notably, although VHSV seems to have a more severe impact on trout farming, it has been observed that the virulence of IHNV has progressively increased in recent years (Toffan personal communications). Indeed, virulence is the result of a complex interplay between epidemiological processes, evolutionary mechanisms and ecological features (Galvani, 2003). Factors such as competition among strains, transmission routes, host adaptation, as well as anthropogenic interventions (e.g., trade and sanitary practices) determine the evolutionary ecology of IHNV and VHSV, all of which clearly require additional investigation.

Molecular epidemiology aims at integrating molecular biology with traditional epidemiology to investigate the mechanisms that shape disease transmission in a susceptible population (Snow, 2011). This strategy has proven to be useful in identifying the origin of disease outbreaks and developing effective control strategies for disease management (Fringuelli et al., 2008; Kristoffersen et al., 2009; Snow, 2011; Lyngstad et al., 2012; Ruane et al., 2015). Herein, we have applied this approach to study the evolutionary and epidemiological dynamics of IHNV and VHSV in Italy, and to identify possible patterns for viral spread. Our phylogeographic analysis revealed no significant association between the phylogenetic clustering of Italian IHNV and VHSV and their water catchment of origin, with the exception of the Po basin, comprising the Mincio and Oglio sub-basins, and the Brenta basin. This result might be explained, at least in part, by the observation that several farms located within the Po basin share the same ownership and, consequently, management practices, and are therefore more likely to suffer disease outbreaks caused by genetically related viral strains. As both IHNV and VHSV are mainly transmitted horizontally (OIE, 2016), it can be assumed that viral diffusion among different trout farms has also occurred through short distance mechanical transmission. This hypothesis is supported by the observation that viruses detected in adjacent trout farms are often genetically nearly identical (i.e., the first epidemiological pattern described above). Passive exposure via water may be a possible route of infection, as previously reported (Gustafson et al., 2007; Viljugrein et al., 2009; Lyngstad et al., 2011, 2012). In particular, Bang Jensen et al. (2014) identified that the distance to the nearest positive farm and the number of upstream farms are significant risk factors for viral spread. It is also noteworthy that neighboring farms might share management operations, thereby facilitating horizontal transmission via fomites (e.g., equipment and vehicles) or through personnel and visitors. Escaping fish from infected farms and piscivorous birds might be also involved as potential viral vectors (Peters and Neukirch, 1986; McAllister and Owens, 1992; St-Hilaire et al., 2002; Lyngstad et al., 2011). While short distance mechanical transmission can explain the first pattern of virus spread, it does not explain the occurrence of genetically distinct strains in neighboring farms nor the presence of identical viruses at facilities located in different water catchments (i.e., second and third epidemiological patterns, respectively). We therefore hypothesize that the spread of the infection might be associated with anthropogenic activities, in which the viruses are dispersed from one farm to the other through trading and management practices. Thus, the transportation of infected fish, eggs or their byproducts for commerce, on growing, processing and stock enhancement as well as co-housing of naïve fish with survivor animals may play an important role in farm-to-farm virus transmission. Notably, movement and trade practices have been previously recognized as a major risk for the introduction of pathogens into fish farms (Troyer and Kurath, 2003; Enzmann et al., 2010; Oidtmann et al., 2011; Reichert et al., 2013; Mardones et al., 2014). In addition, the close genetic relatedness of Italian strains with viruses sampled in other localities highlights that commercial exchanges between different countries might also shape the geographic distribution of IHNV and VHSV and represent a significant risk for viral spread (Enzmann et al., 2010; Reichert et al., 2013).

## Conclusions

This work characterizes the recent molecular evolution and epidemiology of IHNV and VHSV in Italy, in turn helping to reveal the mechanisms that underpin viral emergence and spread. In addition, this study highlights the importance of integrating genetic data with epidemiological information to better understand the transmission pathways of IHNV and VHSV and trace viral spread within populations, which may ultimately assist the design of control strategies for both viral infectious haematopoietic necrosis and viral haemorrhagic septicaemia.

## Author contributions

VP conceived the study and coordinated the work described. AF, EH, AT, GC, and MD contributed to the study design. MA, VP, and AF wrote the manuscript. RQ, MA, AF, and VP performed viral isolation, sequencing and bioinformatics analyses. ChC, ClC, and MD collected epidemiological data and developed geographic maps. EH, AT, GC and MP were involved in the interpretation of the results and critically read the manuscript. All authors read and approved the final manuscript.

## Funding

This work was partially supported by the project MolTraq (ERA-NET EMIDA). ECH is funded by an NHMRC Australia Fellowship (AF30).

### Conflict of interest statement

The authors declare that the research was conducted in the absence of any commercial or financial relationships that could be construed as a potential conflict of interest.
