# Supplementary material for: Molecular Evolution and Phylogeography of Co-circulating IHNV and VHSV in Italy
Source: Front Microbiol. 2016 Aug 23;7:1306. doi: 10.3389/fmicb.2016.01306 (PMC4994472; doi:10.3389/fmicb.2016.01306)
Supplement: Supplementary file 1 [file Table1.DOCX]

Table S1. Values of Association Index (AI). Parsimony Score (PS) and Monophyletic Clade (MC) obtained using BaTS program from the analysis of all the Italian IHNV and VHSV.

| Data set | Statistic | observed  mean | lower 95%  CI | upper 95%  CU | null  mean | lower 95%  CI | upper 95%  CI | P-value |
| --- | --- | --- | --- | --- | --- | --- | --- | --- |
| IHNV | AI | 6.149 | 5.593 | 6.702 | 8.687 | 7.844 | 9.462 | 0 |
|  | PS | 45.057 | 44 | 46 | 57.431 | 54.175 | 60.270 | 0 |
|  | Adige | 1 | 1 | 1 | 1.140 | 1 | 2 | 1 |
|  | Laguna  Venezia | 1 | 1 | 1 | 1.008 | 1 | 1 | 1 |
|  | Livenza | 1.290 | 1 | 2 | 1.139 | 1 | 2 | 1 |
|  | Lemene | 1 | 1 | 1 | 1.037 | 1 | 1.211 | 1 |
|  | Po | 5 | 5 | 5 | 1.658 | 1 | 2.711 | 1.00 × 10^-3^ |
|  | Sile | 4.081 | 4 | 5 | 2.295 | 1.451 | 3.787 | 0.048 |
|  | Piave | 1 | 1 | 1 | 1.067 | 1 | 1.587 | 1 |
|  | Fissero-Tartaro-CanalBianco  Pianura tra Livenza e Piave | 1 | 1 | 1 | 1.005 | 1 | 1 | 1 |
|  | Tagliamento | 1 | 1 | 1 | 1.009 | 1 | 1 | 1 |
|  | Grado-Marano | 1.102 | 1 | 2 | 1.226 | 1 | 2 | 1 |
|  | Brenta | 2.997 | 3 | 3 | 1.221 | 1 | 2 | 0.006 |
| VHSV | AI | 5.163 | 4.427 | 5.881 | 8.183 | 7.268 | 9.034 | 0 |
|  | PS | 41.097 | 39 | 43 | 53.577 | 50.647 | 56.034 | 0 |
|  | Fissero-Tartaro-CanalBianco | 1 | 1 | 1 | 1.005 | 1 | 1 | 1 |
|  | Piave | 2.672 | 2 | 4 | 1.263 | 1 | 2 | 0.053 |
|  | Adige | 1.994 | 2 | 2 | 1.107 | 1 | 1.657 | 0.023 |
|  | Livenza | 1 | 1 | 1 | 1.018 | 1 | 1.093 | 1 |
|  | Lemene | 1 | 1 | 1 | 1.005 | 1 | 1 | 1 |
|  | Po | 13.482 | 12 | 16 | 3.533 | 2.351 | 5.050 | 5.00 × 10^-4^ |
|  | Sile | 3.037 | 3 | 3 | 1.935 | 1.291 | 3.001 | 0.067 |
|  | Grado-Marano | 1.002 | 1 | 1 | 1.144 | 1 | 1.978 | 1 |
|  | Brenta | 1 | 1 | 1 | 1.005 | 1 | 1 | 1 |
